# A Meta-Analysis of the Efficacy and Toxicity of Twice-Daily vs. Once-Daily Concurrent Chemoradiotherapy for Limited-Stage Small Cell Lung Cancer Based on Randomized Controlled Trials

**DOI:** 10.3389/fonc.2019.01460

**Published:** 2020-01-08

**Authors:** Qian Wu, Yiting Xiong, Shujuan Zhang, Xinling Chen, Fengming Yi, Yiping Wei, Wenxiong Zhang

**Affiliations:** ^1^Department of Thoracic Surgery, The Second Affiliated Hospital of Nanchang University, Nanchang, China; ^2^Jiangxi Medical College, Nanchang University, Nanchang, China; ^3^Department of Oncology, The Second Affiliated Hospital of Nanchang University, Nanchang, China

**Keywords:** twice-daily, once-daily, concurrent chemoradiotherapy, limited-stage small cell lung cancer, meta-analysis

## Abstract

**Background:** Currently, the accepted standard management of limited-stage small cell lung cancer (SCLC) is concurrent chemoradiotherapy (CCRT), but the frequency of radiotherapy is controversial. Therefore, this meta-analysis, which compared the efficacy and toxicity between twice-daily (BID) and once-daily (OD) CCRT, was performed to help clinicians make better decisions.

**Methods:** Relevant randomized controlled trials (RCTs) were collected by searching the PubMed, Ovid MEDLINE, Embase, ScienceDirect, Web of Science, the Cochrane Library, Scopus and Google Scholar databases to assess antitumor effects (overall survival, OS; progression-free survival, PFS; overall response rate, ORR) and toxicity (adverse effects, AEs).

**Results:** We screened 1499 articles and included 5 RCTs including 1421 patients. We found that BID CCRT improved OS (hazard ratio, HR = 0.88, 95% confidence interval, CI 0.78–0.99, *p* = 0.03), the 1-year OS rate (OSR-1y, risk ratio, RR = 1.07, 95%CI 1.01–1.13, *p* = 0.03), and OSR-4y (RR = 1.22, 95%CI 1.03–1.43, *p* = 0.02), with better trends in OSR-2y, OSR-3y, and OSR-5y, compared to OD CCRT. In addition, BID CCRT had a higher complete response (CR, RR = 1.31, 95%CI 1.01–1.70, *p* = 0.04) than OD CCRT. PFS (HR = 0.92, 95%CI 0.79–1.07, *p* = 0.29), annual PFS rate, ORR (RR = 0.99, 95%CI 0.93–1.05, *p* = 0.72), and AEs for all grades (RR = 1.00, 95%CI 0.98–1.01, *p* = 0.57), and grades 3–5 (RR = 1.02, 95%CI 0.95–1.09, *p* = 0.60) were similar between the two arms.

**Conclusions:** BID CCRT appears to be better than OD CCRT for limited-stage SCLC, with better antitumor effects (OS, OSR, and CR) and similar AEs. However, the high levels of AEs in both arms should be taken as a sign of caution. More large sample and high-quality RCTs need to be conducted to confirm our conclusions.

## Introduction

It is estimated that 228,150 new cases of lung cancer will be diagnosed in 2019, ~15–30% of which will be small cell lung cancer (SCLC) (1, 2). As reported by the Surveillance, Epidemiology, and End Results (SEER) Cancer Statistics Review, the annual incidence rates of SCLC are decreasing, but the 5-years period survival is only 6.5% (3). This deadly neuroendocrine tumor, which has the characteristics of a short doubling time and early metastasis, is difficult for oncologists to treat (4). For limited-stage SCLC, the current standard treatment is concurrent chemoradiotherapy (CCRT) (5). However, the optimal frequency of CCRT is still controversial.

In light of the National Comprehensive Cancer Network (NCCN) guidelines, twice-daily (BID), and once-daily (OD) CCRT are recommended, but it is not clear which treatment is better (6). Based on the Intergroup 0096 study, 45 Gy in 30 fractions BID CCRT was considered better than 45 Gy in 25 fractions OD CCRT (7). However, this result was not widely accepted because BID CCRT resulted in severe esophagitis and logistical problems (8). The latest large phase III randomized controlled trial (RCT) has reported that the survival outcomes and toxicity are similar between the two regimens (9). Meanwhile, some studies have even shown that OD CCRT improves survival or reduces toxicity compared with BID CCRT (10, 11). To date, the two regimens are both in clinical use with different doses.

As a result, this meta-analysis was performed to compare the efficacy and toxicity of BID CCRT with OD CCRT for limited-stage small cell lung cancer to provide information to clinicians so that they can make better decisions.

## Materials and Methods

We performed this meta-analysis in accordance with the Preferred Reporting Items for Systematic review and Meta-Analysis (Table S1) (12).

### Search Strategy

We searched the following electronic databases: (1) PubMed; (2) Ovid MEDLINE; (3) Embase; (4) ScienceDirect; (5) Web of Science; (6) The Cochrane Library; (7) Scopus; and Google Scholar. The last search was on July 5, 2019. The main search terms were “twice-daily,” “once-daily,” and “small cell lung cancer.” Furthermore, relevant references of the included studies were browsed and manually screened. The retrieval strategies used to search the electronic databases are presented in Table S2.

### Selection Criteria

We set the inclusion criteria based on the principles of PICOS as follows: (1) patient: patients with limited-stage SCLC; according to a 2-stage (limited-stage and extensive-stage) classification scheme, limited-stage SCLC should include patients with a primary tumor and nodal involvement limited to the ipsilateral hemithorax or without distant metastasis (13); (2) intervention and comparison: BID CCRT vs. OD CCRT; (3) outcome: antitumor effect (overall survival, OS; progression-free survival, PFS; overall response rate, ORR), and toxicity (adverse effects, AEs); and (4) study design: RCTs published in English.

We excluded articles without original data, with abstracts only, or with duplicated data as well as reviews, meta-analyses, animal experiments, and unpublished conference papers and clinical trials.

### Data Extraction

The data collected from each included study were general information; characteristics of the study participants; specific modalities of radiation and chemotherapy; survival outcomes (OS, PFS, ORR), including follow-up periods; and AEs, which included the total AEs, all grades and grade 3–5 AEs. The whole information extraction process was completed by two researchers independently, and any disagreements were solved through discussion.

### Quality Assessment

We analyzed the methodological quality of the RCTs according to the Cochrane Handbook, which considers seven dimensions of risk of bias (14). Each dimension contains at least one item, which was used to determine “low risk,” “high risk,” and “risk unknown.” One point was given for “low risk,” and high-quality studies had a score ≥3 points.

We rated the evidence quality for each outcome according to the GRADE (Grading of Recommendations Assessment, Development, and Evaluation) system, as follows: (1) High; (2) Moderate; (3) Low; and (4) Very low (15). The included RCTs may have been degraded by the following five factors: risk of bias, inconsistency, indirectness, imprecision, and publication bias.

### Statistical Analysis

We used Review Manager 5.3 and STATA 12.0 for data analysis. For the time-to-event data, such as OS and PFS, the hazard ratio (HR) with the 95% confidence interval (CI) was used and pooled as an effective indicator (HR > 1 favors the OD arm). If the HR did not appear in the literature, we obtained it from Kaplan–Meier curves through the method described by Tierney et al. (16). In addition, for dichotomous outcomes, such as ORR and AEs, we calculated and pooled risk ratios (RR) with the 95% CI (ORR: RR > 1 favors the BID arm; AEs: RR > 1 favors the OD arm). Subgroup analyses of OS and PFS were carried out for the continent, models of CCRT regarding the radiotherapy dose (fractional and full) and timing, and chemotherapy cycle. The fixed effect model was initially used. χ^2^ tests and the *I*^2^ index were used to investigate heterogeneity among trials. When substantial heterogeneity appeared (*I*^2^ > 50% or *p* < 0.1 for the χ^2^ test), the random effect model was used and sensitivity analysis was conducted (14). Publication bias detection was performed for OS and PFS. *P* < 0.05 indicated a significant difference.

## Results

### Search Results and Study Quality Assessment

**Figure 1:**
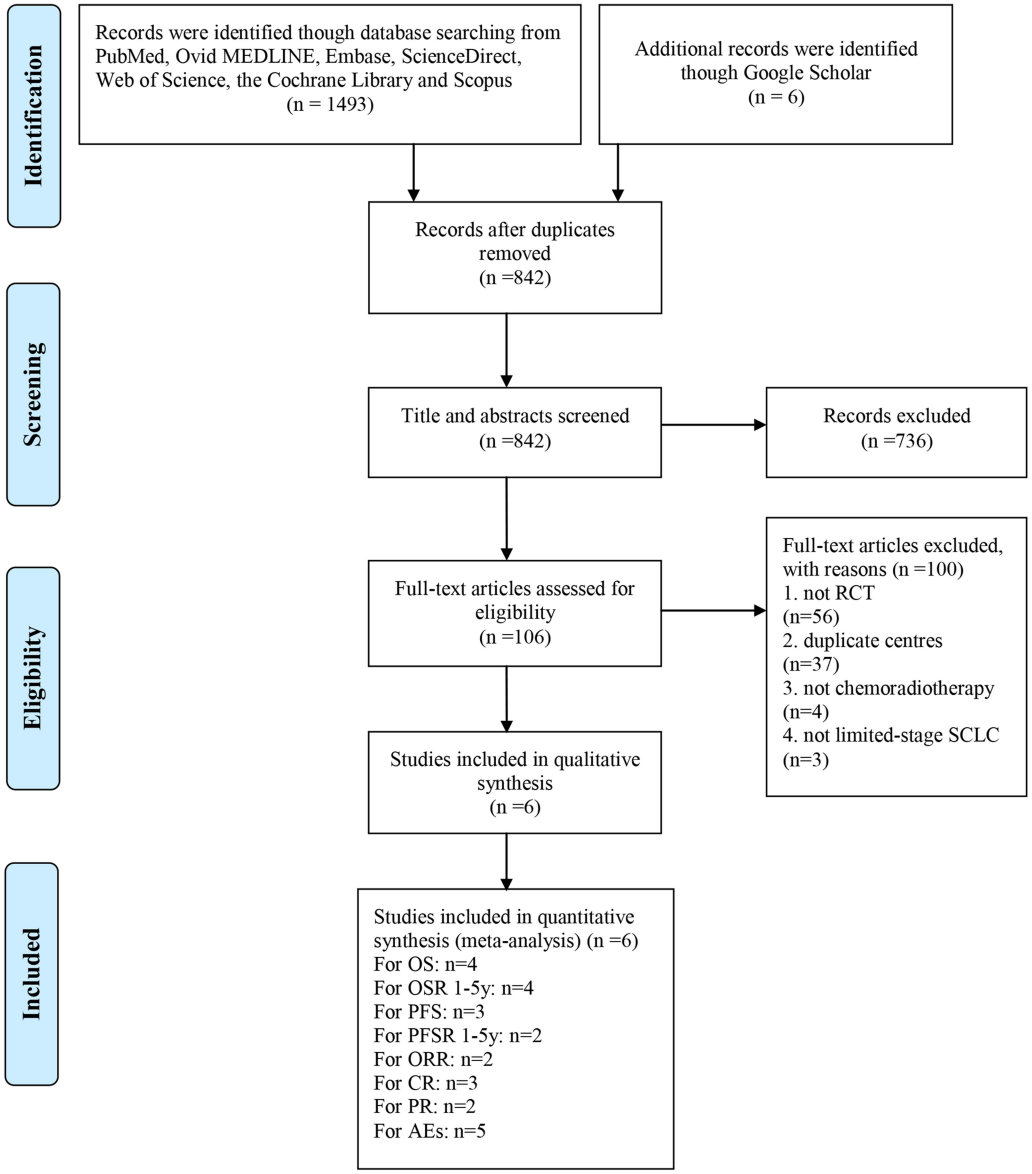
Flow chart of literature selection.

A total of 1499 studies were identified from the search of the electronic databases, and six related articles based on five RCTs including 1,421 patients were eligible for inclusion (7, 9, 17–20). Bonner et al. (17) and Schild et al. (18) described the same study and different results. The former described AEs and the response rate, and the latter described survival outcomes. The literature selection is shown in Figure 1. All of the studies were of high quality. The detailed quality assessment of each included study is shown in Figure S1. The evidence quality of 24/46 outcomes was “High,” and the rest was “Moderate” (Table S3).

### Basic Characteristics of the Studies

Of the five studies, two were conducted in America (7, 17, 18), one in Norway (20), one in England (19), and one with patients from multiple centers from eight countries (9). Eligible patients included in these trials were required to have a good performance status (the performance score was recorded as 0–2) and a limited-stage disease that met the above definition. The mode of CCRT was not identical in each experiment. Physical dose was converted into equivalent dose in 2 Gy/f (EQD2) linear quadratic model, with alpha/beta = 10 for early reaction tissues (tumor tissue) and alpha/beta = 3 for late reaction tissues (normal tissue), according to the method provided by Fowler (21). Patients who achieved complete response (CR), which referred to the complete disappearance of the tumor in this meta-analysis, received prophylactic cranial irradiation (PCI). The specific basic characteristics of the studies are presented in Table 1.

**Table 1:** Basic characteristics of included literatures.

**Study**			**Country**	**Patient (*****n*****)**		**Median age (year)**		**Gender M/F (*****n*****)**		**Radiotherapy dose**			**Radiotherapy timing**	**Chemotherapy scheme**	**Median follow-up (months)**
				**BID**	**OD**	**BID**	**OD**	**BID**	**OD**	**Physical dose**	**${\text{D}}_{\text{E}}^{\text{a}}$ (Gy)**	**${\text{D}}_{\text{L}}^{\text{b}}$ (Gy)**			
NCCTG	1999	Bonner et al. (17)	America	130	132	-	-	74/56	77/55	48Gy/1.5Gy/32F/3W;	46.0;	43.2;	W13	EP: W1,5,9,13,17,21	39
										50.4Gy/1.8Gy/28F/6W	49.6	48.4			
NCCTG	2004	Schild et al. (18)	America	130	131	62.5	63	74/56	76/55			Same as above			88.8
ECOG	1999	Turrisi et al. (7)	America	211	206	61	63	58/42	59/41	45Gy/1.5Gy/30F/3W;	43.1;	40.5;	W1	EP: W1,4,7,10	96
										45Gy/1.8Gy/25F/5W	44.2	43.2			
CNFT	2012	Colaco et al. (19)	UK	12	26	61		–/–	–/–	45Gy/1.5Gy/30F/3W;	43.1;	40.5;	W4;	EP: W1,4,7,10	16.9
										66Gy/2.0Gy/33F/6W	66	66			
RHA	2016	Grønberg et al. (20)	Norway	73	84	63	63	36/37	45/39	45Gy/1.5Gy/30F/3W;	43.1;	40.5;	W4	EP: W1,4,7,10	81
										42Gy/2.8Gy/15F/3W	44.8	48.7			
CONVERT	2017	Faivre-Finn et al. (9)	Multiple	274	273	62	63	147/127	150/123	45Gy/1.5Gy/30F/3W;	43.1;	40.5;	W4;	EP: W1,4,7,10,13,16	45
										66Gy/2.0Gy/33F/7W	66	66			

*BID, twice-daily; OD, once-daily; M, male; F, female; D, days; W, week(s); OS, overall survival; PFS, progression-free survival; Gy, grays; F(f), fraction(s); EP, etoposide/cisplatin*.

^a^D_E_ (Equivalent dose in 2 Gy/f of tumor tissue) = full dose*(fractional dose+10)/(2+10).

^b^D_L_ (Equivalent dose in 2 Gy/f of normal tissue) = full dose*(fractional dose+3)/(2+3).

### Survival Outcomes

**Figure 2:**
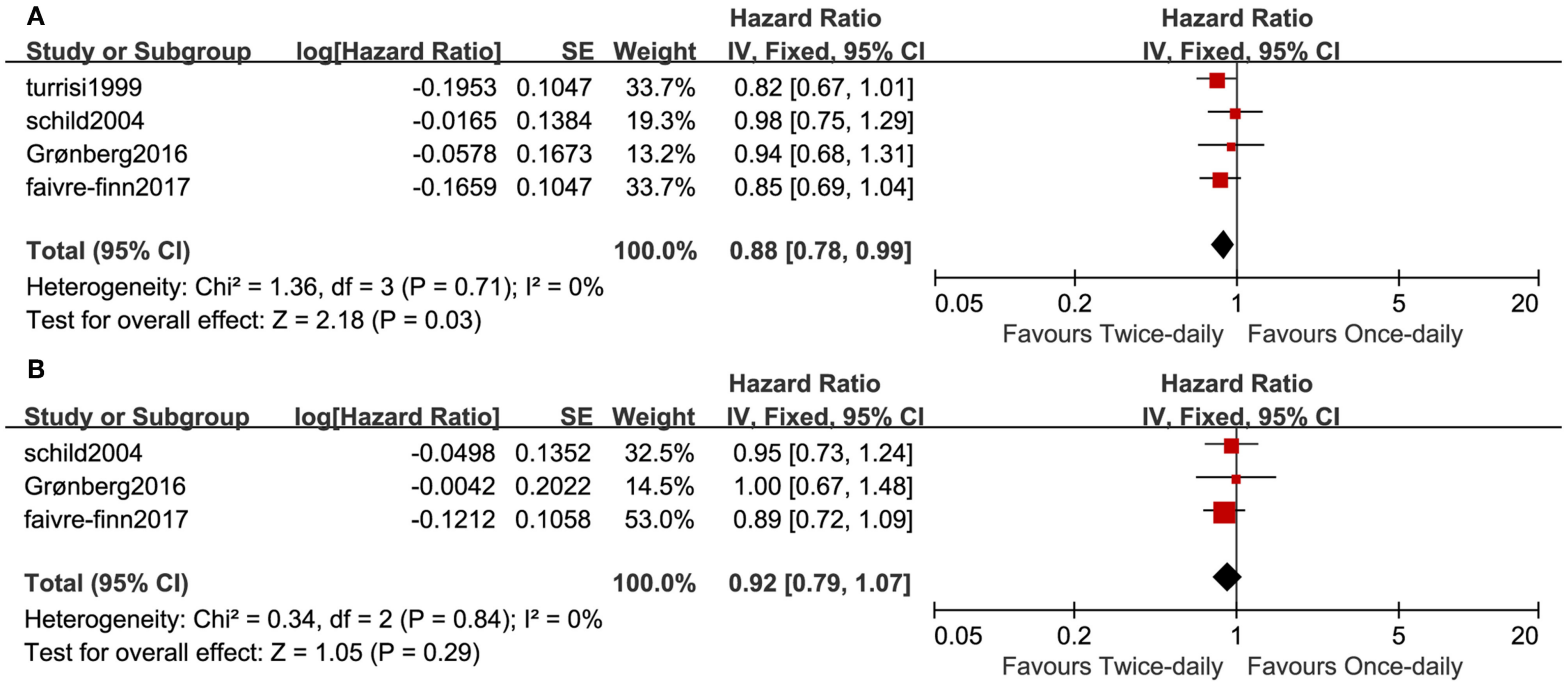
Forest plots of OS **(A)** and PFS **(B)**.

**Figure 3:**
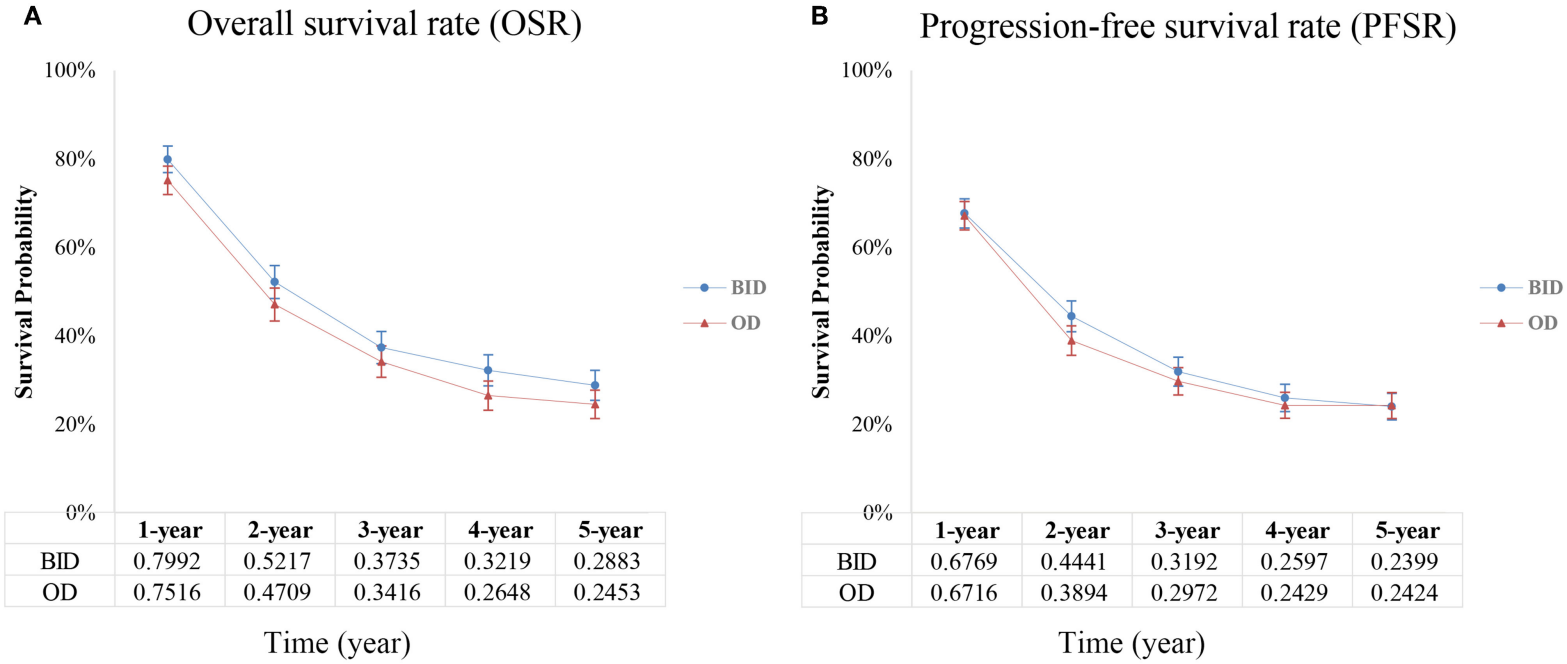
Annual OSR **(A)** and annual PFSR **(B)**.

Four studies compared OS in the two arms and included 1,378 patients (687 for the twice-daily group and 691 for the once-daily group). We found that BID CCRT led to better OS (HR = 0.88, 95%CI 0.78–0.99, *p* = 0.03, *I*^2^ = 0%; Figure 2A) and increased median OS by ~4 months (25.6 months vs. 21.6 months) vs. OD CCRT. The BID arm had a higher 1-year overall survival rate (OSR-1y, RR = 1.07, 95%CI 1.01–1.13, *p* = 0.03, *I*^2^ = 3%) and OSR-4y (RR = 1.22, 95%CI 1.03–1.43, *p* = 0.02, *I*^2^ = 0%), and OSR-2y, OSR-3y and OSR-5y tended to favor the BID arm compared with the OD arm (Figure 3A and Figure S2).

Three studies analyzed PFS. We found that PFS was similar (HR = 1.08, 95%CI 0.93–1.25, *p* = 0.30, *I*^2^ = 0%; Figure 2B) between the two arms. The annual progression-free survival rate (PFSR) within 5 years tended to favor the BID arm but without a significant difference (Figure 3B and Figure S3).

**Figure 4:**
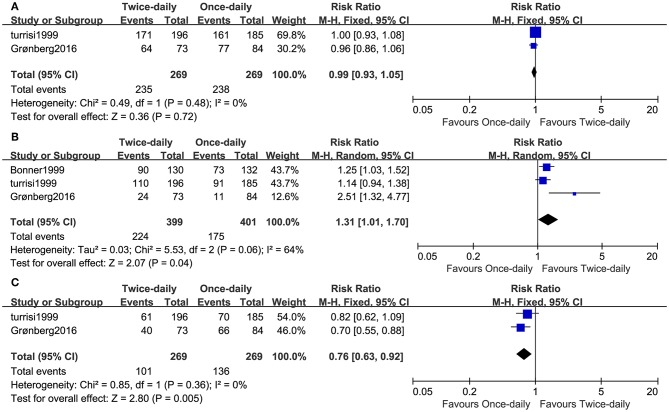
Forest plots of ORR **(A)**, CR **(B)**, and PR **(C)**.

Two studies including 538 patients reported the ORR. We found that the ORR was similar (RR = 0.99, 95%CI 0.93–1.05, *p* = 0.72, *I*^2^ = 0%; Figure 4A) and high (BID: 87% vs. OD: 88%) between the two arms. Three studies compared CR, while two studies compared partial response (PR). The BID arm had higher CR (RR = 1.31, 95%CI 1.01–1.70, *p* = 0.04, *I*^2^ = 64%; Figure 4B) and lower PR (RR = 0.76, 95%CI 0.63–0.92, *p* = 0.005, *I*^2^ = 0%; Figure 4C) compared with the OD arm.

### Toxicity

Two studies compared all grades of AEs. We found that the total AEs (RR = 1.00, 95%CI 0.98–1.01, *p* = 0.57) and the ten most reported AEs (myelotoxicity, leukopenia, fatigue, neutropenia, anemia, esophagitis, nausea, thrombocytopenia, weight loss, and anorexia) were similar between the two arms (Table 2).

**Table 2:** Top 10 adverse effects (all grades).

**All grades adverse effects**	**Twice-daily arm (event/total)**	**Once-daily arm (event/total)**	**The incidence of adverse effects (%)^**a**^**	**RR (95% CI)**	***P-*value**	**Heterogeneity**	
						***I*^**2**^ (%)**	***P-*value**
Total	204/206	202/203	99.27	1.00 [0.98, 1.01]	0.57	-	-
Myelotoxicity^b^	199/206	201/203	97.80	0.98 [0.95, 1.00]	0.10	-	-
Leukopenia	197/206	190/203	94.62	1.02 [0.98, 1.07]	0.36	-	-
Fatigue	243/266	247/263	92.63	0.97 [0.93, 1.02]	0.26	-	-
Neutropenia	421/472	396/466	87.10	1.05[1.00, 1.10]	0.05	0	0.36
Anemia	401/472	390/466	84.33	1.02 [0.96, 1.07]	0.59	0	0.70
Esophagitis	365/460	315/499	74.81	1.19 [0.75, 1.90]	0.46	96	<0.00001
Nausea	195/266	197/263	74.10	0.98 [0.88, 1.08]	0.68	-	-
Thrombocytopenia	134/206	125/203	63.33	1.06 [0.91, 1.22]	0.47	-	-
Weight loss	136/206	118/203	62.10	1.14 [0.98, 1.32]	0.10	-	-
Anorexia	153/266	150/263	57.28	1.01 [0.87, 1.17]	0.91	-	-

*RR, risk ratio; CI, confidence interval*.

a *Event (twice-daily arm + once-daily arm)/Total (twice-daily arm + once-daily arm)*.

b *Myelotoxicity was defined as any decrease in marrow-derived cells in the peripheral-blood counts*.

Five studies including 1,366 patients analyzed grade 3–5 AEs. We found that the total AEs (RR = 1.17, 95%CI 0.80–1.72, *p* = 0.42) and the 10 most reported AEs (myelotoxicity, leukopenia, neutropenia, thrombocytopenia, esophagitis, anemia, infection, fatigue, and vomiting) were similar between the two arms (Table 3).

**Table 3:** Top 10 adverse effects (grade 3–5).

**Grade 3–5 adverse effects**	**Twice-daily arm (event/total)**	**Once-daily arm (event/total)**	**The incidence of adverse effects (%)^**a**^**	**RR (95% CI)**	***P-*value**	**Heterogeneity**	
						***I*^**2**^ (%)**	***P-*value**
Total	255/336	230/335	72.28	1.17 [0.80, 1.72]	0.42	87	0.005
Myelotoxicity^b^	179/206	173/203	86.06	1.02 [0.94, 1.10]	0.63	-	-
Leukopenia	340/409	334/419	81.40	1.04 [0.98, 1.11]	0.23	0	0.61
Neutropenia	422/545	395/550	74.61	1.06 [0.95, 1.17]	0.30	57	0.1
Thrombocytopenia	129/409	155/419	34.30	0.86 [0.72, 1.03]	0.10	24	0.27
Esophagitis	157/675	121/691	20.35	1.39 [0.91, 2.11]	0.13	67	0.002
Anemia	109/675	100/682	15.40	1.10[0.86, 1.40]	0.46	11	0.34
Infection	91/675	97/682	13.71	0.99[0.77, 1.26]	0.93	0	0.72
Nausea	45/396	48/395	11.76	0.94[0.60, 1.37]	0.74	0	0.70
Fatigue	31/266	31/263	11.72	0.99 [0.62, 1.58]	0.96	-	-
Vomiting	52/602	50/598	8.50	1.04[0.72, 1.50]	0.85	0	0.84

*RR, risk ratio; CI, confidence interval*.

a *Event (twice-daily arm + once-daily arm)/Total (twice-daily arm + once-daily arm)*.

b *Myelotoxicity was defined as any decrease in marrow-derived cells in the peripheral-blood counts*.

Four studies compared the treatment-related mortality (TRM). We found that the TRM (RR = 0.96, 95%CI 0.50–1.87, *p* = 0.92, *I*^2^ = 33%; Figure S4A) was similar between the two arms. The top three causes of treatment-related deaths were pulmonary effects, infection and neutropenic sepsis, and no significant difference was found between the two arms (Table S4).

### Subgroup Analysis

To explore whether the survival outcomes changed, we established subgroups for OS and PFS according to continent, radiotherapy dose (fractional and full) and timing, and chemotherapy cycle. We found that BID CCRT led to better OS vs. OD CCRT in standard fractionation (1.8 or 2 Gy/f, HR = 0.87, 95%CI 0.76–0.98, *p* = 0.03, *I*^2^ = 0%). The results of other subgroups tended to support BID CCRT compared with OD CCRT although no significant differences were found (Table 4).

**Table 4:** Subgroup analysis for overall survival and progression-free survival.

**Subgroup**	**OS**				**PFS**			
	**No.of studies**	**HR (95% CI)**	***P***	***I*^**2**^ (%)**	**No.of studies**	**HR (95% CI)**	***P***	***I*^**2**^ (%)**
**Total**	4	0.88 [0.78, 0.99]	0.03	0	3	0.92 [0.79, 1.07]	0.29	0
**Continent**								
North America	2	0.88 [0.75, 1.03]	0.12	6	1	0.95 [0.73, 1.24]	0.71	-
Europe	1	0.94 [0.68, 1.31]	0.73	-	1	1.00 [0.67, 1.48]	0.98	-
**Radiotherapy fractional dose**								
1.5Gy (BID) vs. 1.8Gy/2.0Gy (OD)	3	0.87 [0.76, 0.98]	0.03	0	2	0.91 [0.77, 1.07]	0.26	0
1.5Gy (BID) vs. 2.8Gy (OD)	1	0.94 [0.68, 1.31]	0.73	-	1	1.00 [0.67, 1.48]	0.98	-
**Equivalent dose in 2 Gy/f**								
< 60 Gy	3	0.89 [0.77, 1.03]	0.55	0	2	0.96 [0.77, 1.20]	0.75	0
≥60 Gy	1	0.85 [0.69, 1.04]	0.11	-	1	0.89 [0.72, 1.09]	0.25	-
**Radiotherapy fractional dose**								
**Radiotherapy timing**								
The first cycle of chemotherapy	1	0.82 [0.67, 1.01]	0.06	-	1	0.89 [0.72, 1.09]	0.25	-
The second cycle of chemotherapy	2	0.87 [0.73, 1.04]	0.13	0	2	0.91 [0.76, 1.09]	0.31	0
The fourth cycle of chemotherapy	1	0.98 [0.75, 1.29]	0.91	-	1	0.95 [0.73, 1.24]	0.71	0
**Chemotherapy cycle**								
Four cycles	2	0.86 [0.72, 1.02]	0.08	0	1	1.00 [0.67, 1.48]	0.98	-
Six cycles	2	0.89 [0.76, 1.05]	0.18	0	2	0.91 [0.77, 1.07]	0.26	0

*OS, overall survival; PFS, progression-free survival; HR, hazard ratio; CI, confidence interval; Gy, grays; BID, twice-daily; OD, once-daily; f, fraction*.

### Sensitivity Analysis

To evaluate sensitivity and stability, we conducted a sensitivity analysis for CR with significant heterogeneity. We found that the outcome of CR was stable after omitting one study (Figure S5).

### Publication Bias

We found no publication bias for OS (Begg's test *p* = 0.734; Egger's test *p* = 0.160) and PFS (Begg's test *p* = 0.296; Egger's test *p* = 0.205) in this meta-analysis (Figure S6).

## Discussion

The median survival of limited-stage SCLC has been reported to be 15–20 months, indicating that this cancer extremely malignant (22). For limited-stage SCLC that is not suitable for surgery, the accepted standard treatment is CCRT (23). However, there is much debate about whether radiotherapy should be BID or OD (24). This is the first meta-analysis to determine the optimal scheme between the two regimens; this meta-analysis includes five RCTs including 1,421 patients. The results of this meta-analysis indicated that BID CCRT improved OS, OSR-1y, and OSR-4y, with better trends in OSR-2y, OSR-3y, and OSR-5y, compared to OD CCRT. Subgroup analyses of OS in standard fractionation also supported this result. In addition, BID CCRT had a higher complete response than OD CCRT. No significant differences were found for PFS, annual PFSR, ORR, or AEs of grade 3–5 or all grades between the two arms. Other subgroup analyses of OS and PFS regarding continent, radiotherapy dose (fractional and full) and timing, and chemotherapy cycle tended to favor BID CCRT but without significant differences between the two arms.

In fact, Turrisi et al. reported that the OS of BID CCRT was better than that of OD CCRT (*p* = 0.04), which agreed with the results of this meta-analysis (median OS: 25.6 months vs. 21.6 months) (7). The annual OSR suggested that there was a potential survival advantage in BID CCRT compared to OD CCRT. Schreiber et al. also found that compared with OD CCRT with different doses, the median survival time of 45 Gy BID CCRT was longer (BID: 22.1 months vs. OD: 45 Gy, 17.2 months, 46–59.4 Gy, 18.3 months, 60–61.2 Gy, 19.2 months, and 62–72 Gy, 19.5 months) (25). However, a cohort study in Asia found that OD CCRT had a significantly longer median OS than BID CCRT (47.2 months vs. 32.8 months) (10). The studies we included in this meta-analysis mainly focused on Europe and the Americas, and the results of this meta-analysis may only apply in these areas. Subgroup analyses of OS in standard fractionation indicated BID CCRT was superior to OD CCRT but no significant difference was found in hypofractionation (>2 Gy/f) and other subgroup analysis results, which might be caused by an insufficient sample size. Xia et al. study also shown that 2.5 Gy/f OD CCRT had favorable survival (26). The ongoing CALGB 30610/RTOG 0538 study (NCT00632853) is further exploring the effects of high dose fractions (27). According to the NCCN guidelines, which recommend the first or second cycle of chemotherapy be combined with radiotherapy, we performed a set of subgroup analyses and found that OS was improved in the BID arm when radiotherapy was initiated from the first or second cycle of chemotherapy vs. the OD arm (HR = 0.85, 95%CI 0.75–0.97, *p* = 0.02). One possible reason for this improvement is that repopulation of tumor cells is accelerated during chemotherapy cycles and proliferating cells may be more sensitive to BID irradiation (28). Another possible reason for the improvement is that lower overall doses lead to improved treatment delivery of BID CCRT compared with OD CCRT; thus, more patients receive full-dose irradiation. The PFS and PFSR results were consistent with the included studies, which tended to favor BID CCRT but without statistically significant differences compared with OD CCRT. The subgroup analysis reported similar results. However, a previous cohort study reported that the median PFS was longer in the OD arm vs. the BID arm (20.1 months vs. 18.8 months), although there was no significant difference. We recommend that high-quality and well-designed RCTs should take the Asian region into account.

In all of the included studies, the ORRs to CCRT of the two arms were both nearly 90 percent, and no significant difference was reported, which formed the basis of our results. Kubota et al. also found that 95 percent of limited-stage SCLC patients receiving BID CCRT combined with etoposide and cisplatin had an objective response in a large sample RCT (29). In general, for treatment of a tumor with poor prognosis, the CR and PR results should support the same arm (30). However, in this meta-analysis, the higher CR and lower PR in the BID regime only demonstrated an advantage due to the high ORR. These results are in line with the RHA study and NCCTG study but not the ECOG study, in which the difference between the two arms was not significant. Salama et al. found low CR (41%) and high PR (47%) in connected Cancer and Leukemia Group B limited-stage SCLC trials (39808, 30002, and 30206) using high dose OD CCRT, which was similar to our results (OD: 44% and 51%) (31). It was possible that BID CCRT provided a higher biological effective dose for a shorter time of radiation compared with the OD arm, thus preventing further tumor replication.

Regarding AEs, although no significant difference was reported between the two arms in most of the included studies, the NCCTG study found that BID CCRT had a higher incidence of grade 3–5 AEs (54 vs. 39%) and thrombocytopenia (45 vs. 59%) and the ECOG study found that BID CCRT had a higher incidence of esophagitis (63 vs. 44%) vs. OD CCRT. Thus, we conducted a subgroup analysis of grade 3–5 esophagitis and found similar results in the subgroup from North America, where the included studies were published in 1999 (RR = 2.06, 95%CI 1.46–2.89, *p* < 0.0001; Figure S4B). However, a recent study reported that the incidence of severe esophagitis after BID CCRT was only 2% (29). A possible reason for this result is that previous studies used large and 2-dimensional fields with elective nodal irradiation, which increased radiation exposure to normal tissues (9). At present, 3D- or intensity-modulated radiotherapy provides fewer, higher dose fractions and achieves similar results as the hyper-fractionated regimen (32). We suggest that future studies should use advanced radiotherapy techniques to determine the optimal dose and fractionation for limited-stage SCLC therapy.

There were several limitations of this meta-analysis: (1) Only five RCTs were included, which impacted the representativeness of the results; (2) substantial heterogeneity appeared in the analysis which might make the results unstable: most of the participants were from Europe and North America, which affected the representativeness of the results; only the CONVERT study reported similar improvements in OS in elderly patients for both regimes, which might not allow the elimination of the interference of age (33); PCI might be unbalanced between the two arms (RR = 1.07, 95%CI 1.00–1.14, *p* = 0.06, *I*^2^ = 0%; Figure S4C), which had been reported to improve OS (34); at baseline, all patients received CT scans, and only some patients from CONVERT received PET/CT imaging. In fact, pretreatment with PET scans had been reported to improve OS (35); only the RHA study had a definite staging on CT scans before admission, and the rest were performed later, which might lead to stage migration; (3) some of the time-to-event data were obtained from Kaplan–Meier curves, which were unstable; factors such as necrosis and hypoxia could affect the alpha/beta value, which made the inaccuracy of the formula to estimate EQD2 increase; and (4) there is an economic argument for BID CCRT as compared to OD CCRT because of the shortened treatment time, but no formal economic analysis had been done on both regimens. These findings suggest that further well-designed prospective studies need to be carried out.

## Conclusion

BID CCRT appears to be better than OD CCRT, with better antitumor effects (OS, OSR, and CR) and similar AEs for limited-stage SCLC patients. However, the high levels of AEs in both regimens should be a sign of caution. Subgroup analysis of OS and PFS tended to favor BID CCRT, especially comparing OD CCRT in standard fractionation. Moreover, more large sample and well-designed RCTs need to be conducted to confirm our conclusions.

## Data Availability Statement

All datasets generated for this study are included in the article/Supplementary Material.

## Author Contributions

QW: full access to all of the data in the manuscript and takes responsibility for the integrity of the data, the accuracy of the data analysis, and statistical analysis. QW and WZ: drafting of the manuscript. QW, WZ, YX, SZ, XC, and FY: critical revision of the manuscript for important intellectual content. YW and WZ: supervision. All authors: concept and design and acquisition, analysis, or interpretation of data.

### Conflict of Interest

The authors declare that the research was conducted in the absence of any commercial or financial relationships that could be construed as a potential conflict of interest.

## Supplementary Material

Figure S1 Risk of bias of included literatures.

Figure S2 Forest plot of annual OSR.

Figure S3 Forest plot of annual PFSR.

Figure S4 Forest plots of TRM **(A)**, grade 3–5 esophagitis **(B)**, and PCI **(C)**.

Figure S5 Sensitivity analysis about CR.

Figure S6 Egger tests about OS **(A)** and PFS **(B)**.

Table S1 PRISMA checklist.

Table S2 Search strategies of electronic databases.

Table S3 GRADE quality assessment by therapeutic strategy and study design for the outcomes of survival and toxicity.

Table S4 Ranking of the cause of treatment-related mortality.

## Abbreviations

SCLC: small cell lung cancer
SEER: Surveillance, Epidemiology, and End Results
CCRT: concurrent chemoradiotherapy
NCCN: National Comprehensive Cancer Network
BID: twice-daily
OD: once-daily
Gy: gray
RCT: randomized controlled trial
PRISMA-P: Preferred Reporting Items for Systematic review and Meta-Analysis Protocols
OS: overall survival
PFS: progression-free survival
ORR: objective response rate
AEs: adverse effects
GRADE: Grading of Recommendations Assessment, Development, and Evaluation
HR: hazard ratio
CI: confidence interval
RR: risk ratio
f: fraction
EQD2: equivalent dose in 2 Gy/f
CR: complete response
PCI: prophylactic cranial irradiation
OSR: overall survival rate
y: year
PFSR: progression-free survival rate
PR: partial response
TRM: treatment-related mortality.
