# Supplementary figures and images for: A Meta-Analysis of the Efficacy and Toxicity of Twice-Daily vs. Once-Daily Concurrent Chemoradiotherapy for Limited-Stage Small Cell Lung Cancer Based on Randomized Controlled Trials
Source: Front Oncol. 2020 Jan 8;9:1460. doi: 10.3389/fonc.2019.01460 (PMC6960125; doi:10.3389/fonc.2019.01460)

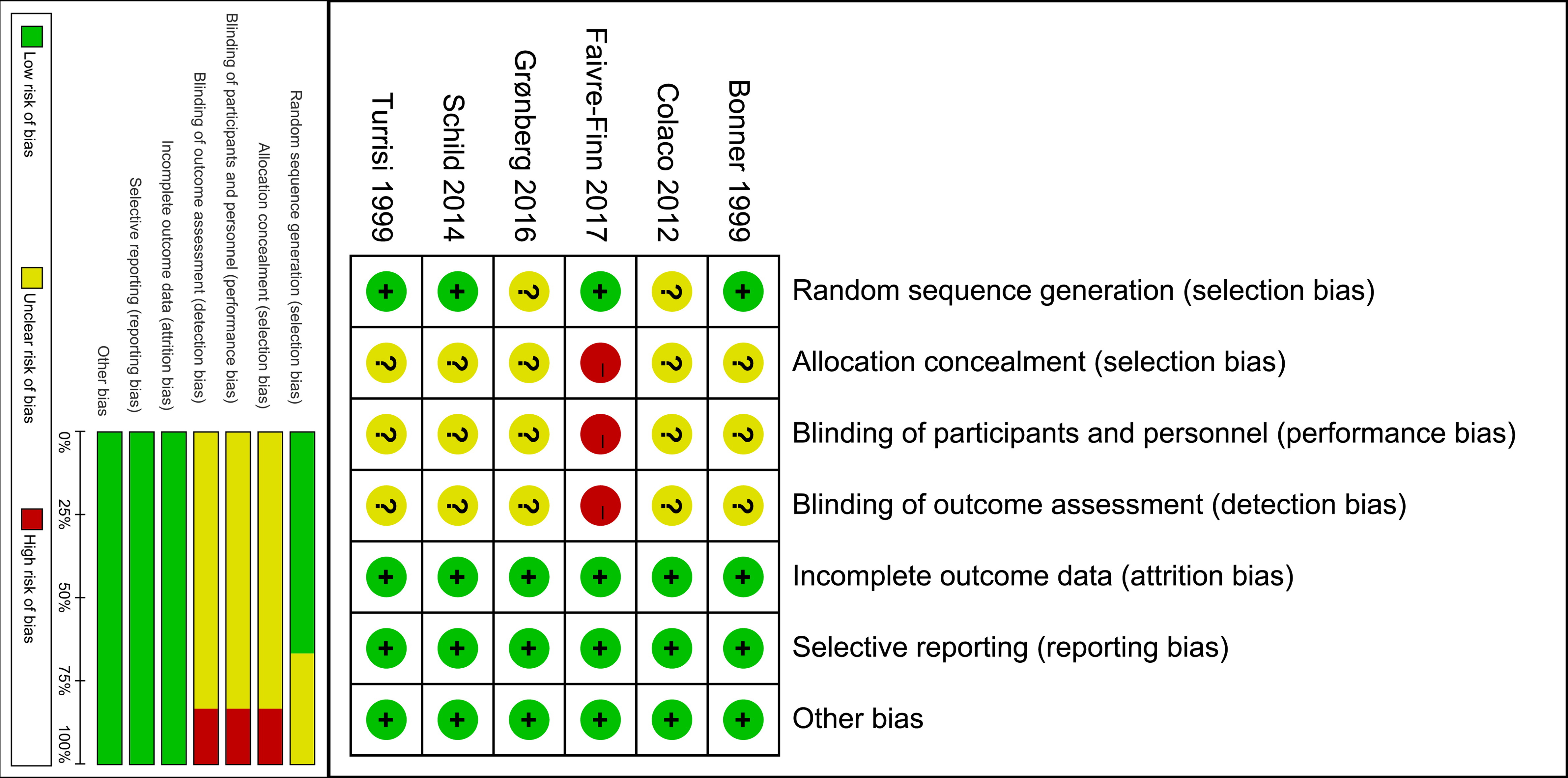

Supplement: Figure S1 — Risk of bias of included literatures. [file Image_1.TIF]

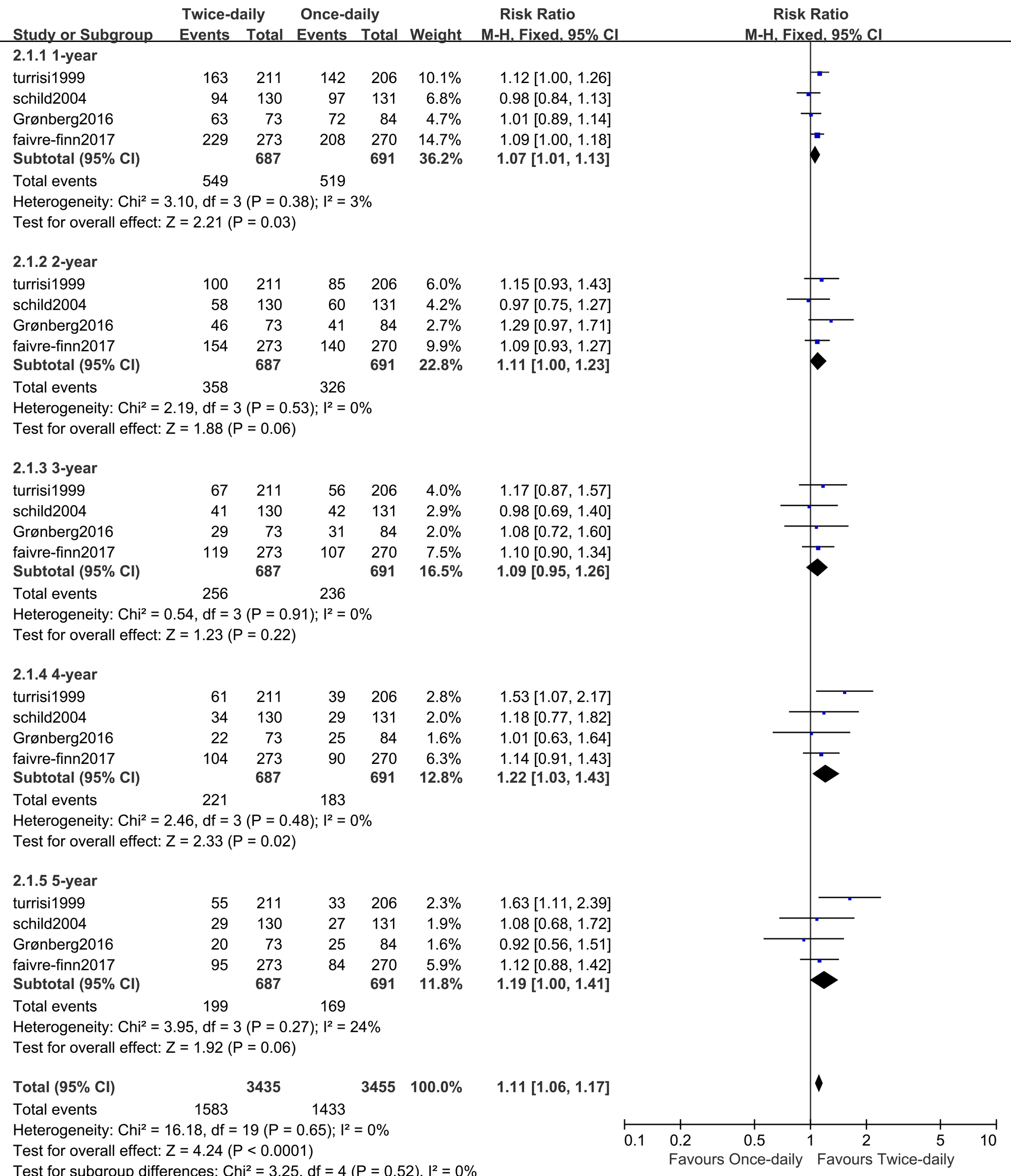

Supplement: Figure S2 — Forest plot of annual OSR. [file Image_2.TIF]

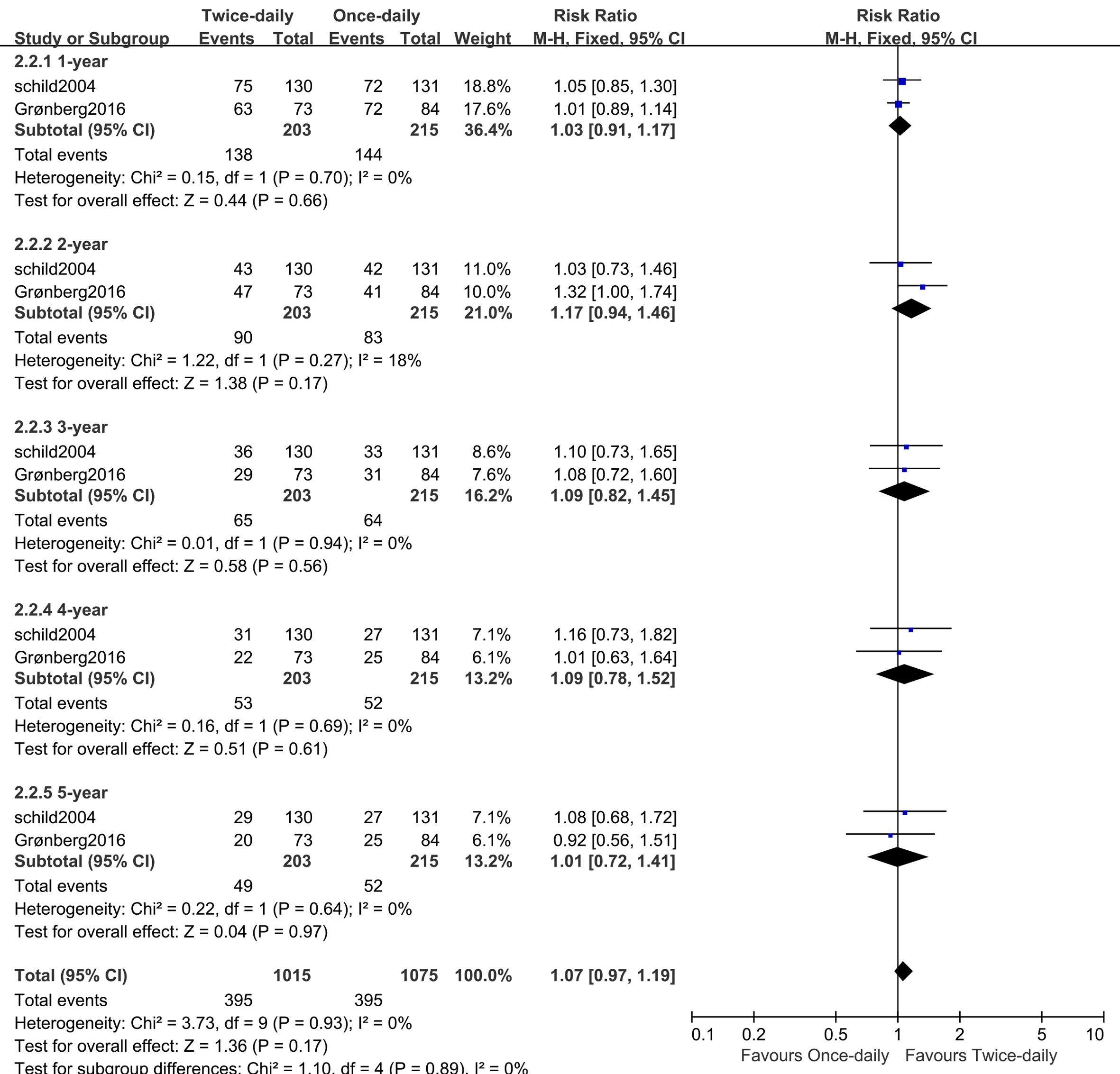

Supplement: Figure S3 — Forest plot of annual PFSR. [file Image_3.TIF]

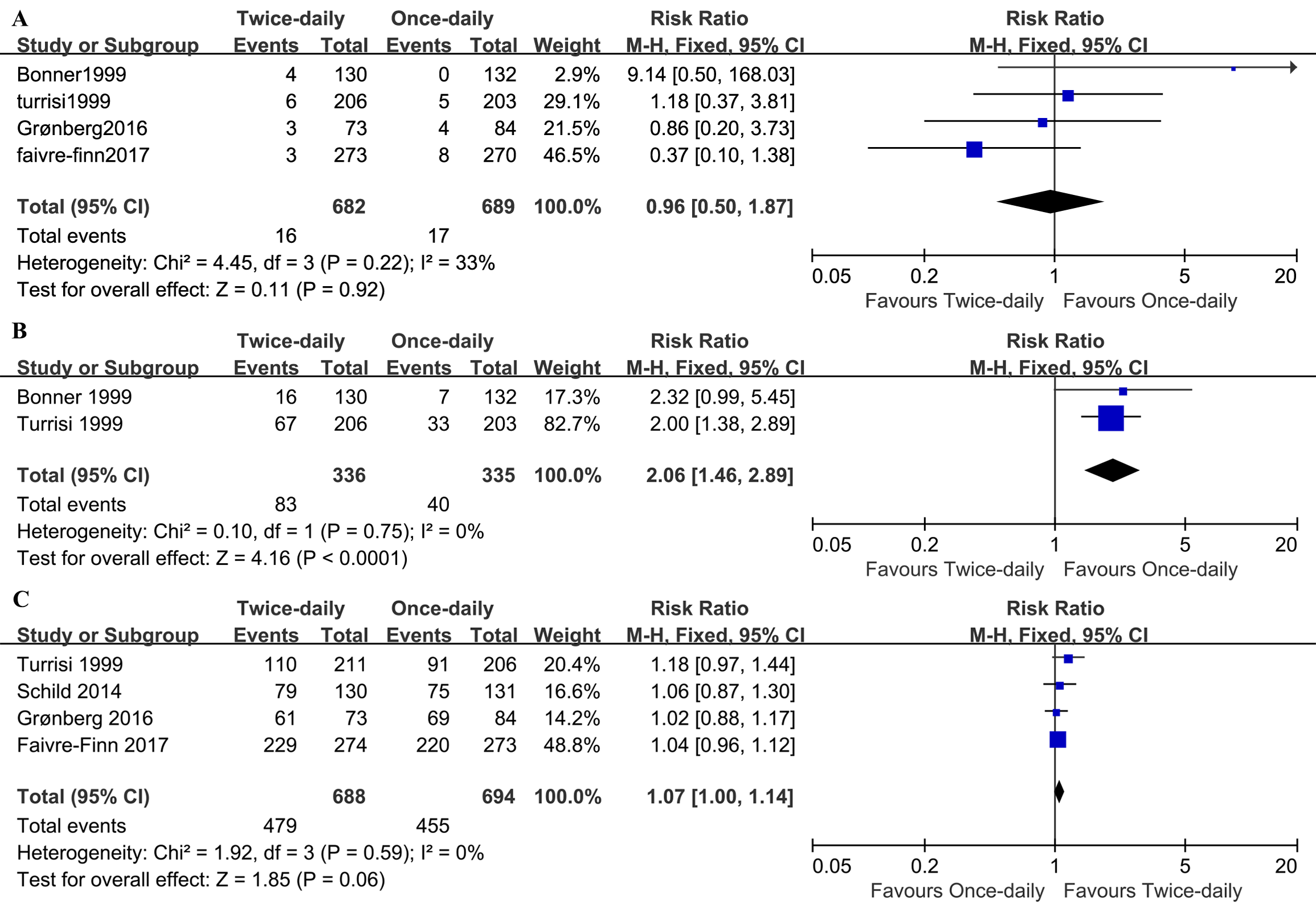

Supplement: Figure S4 — Forest plots of TRM (A), grade 3–5 esophagitis (B), and PCI (C). [file Image_4.TIF]

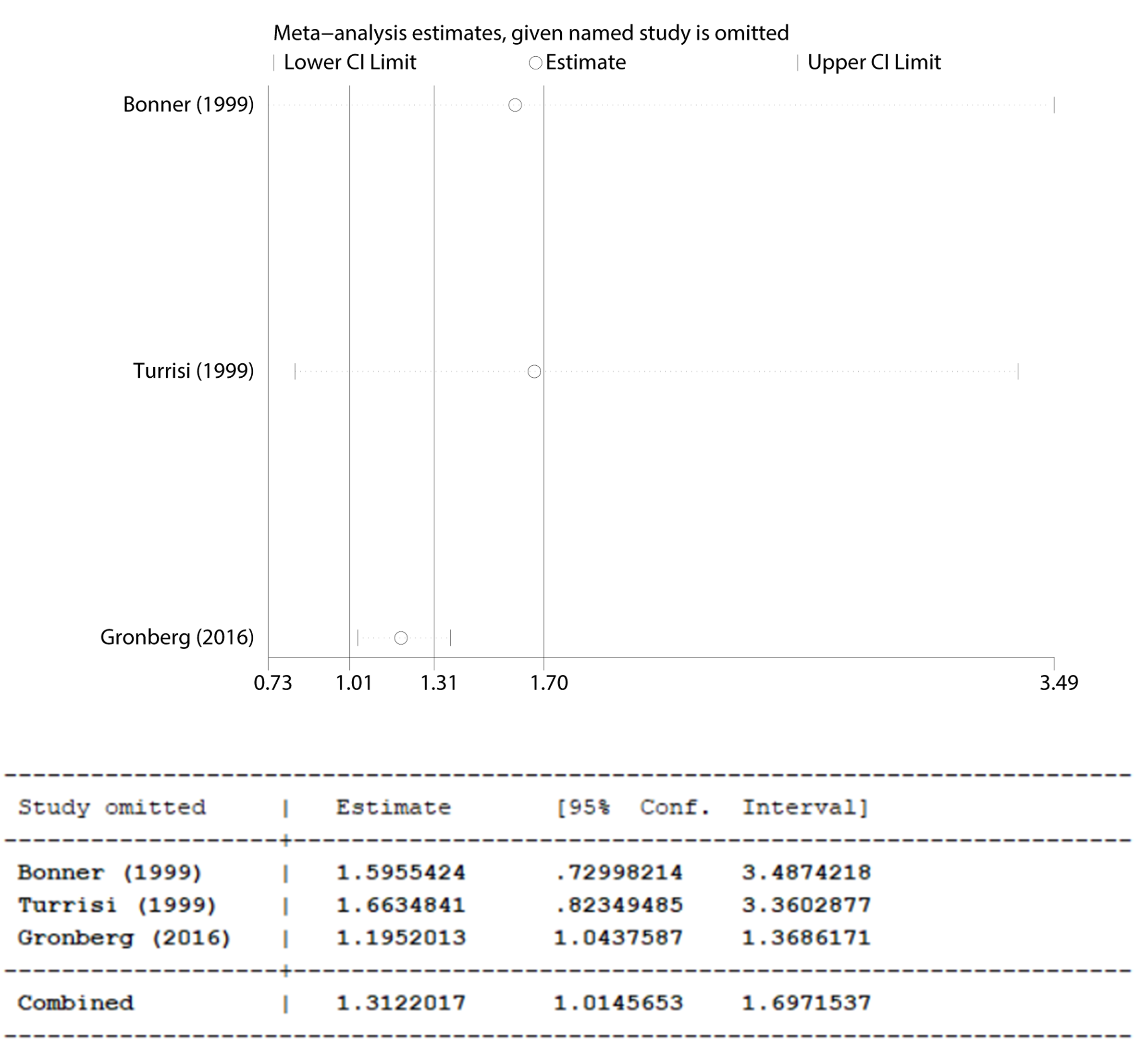

Supplement: Figure S5 — Sensitivity analysis about CR. [file Image_5.TIF]

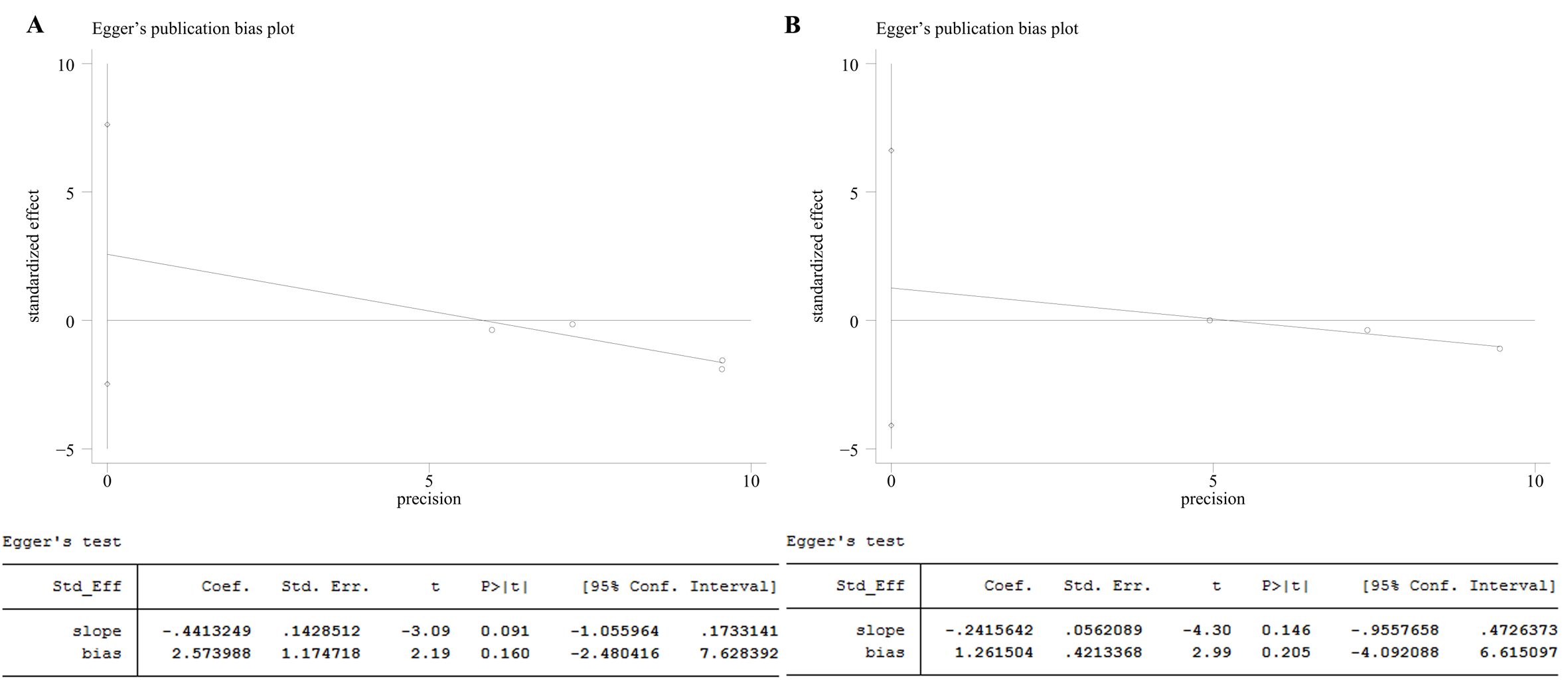

Supplement: Figure S6 — Egger tests about OS (A) and PFS (B). [file Image_6.TIF]
