# Supplementary material for: A Meta-Analysis of the Efficacy and Toxicity of Twice-Daily vs. Once-Daily Concurrent Chemoradiotherapy for Limited-Stage Small Cell Lung Cancer Based on Randomized Controlled Trials
Source: Front Oncol. 2020 Jan 8;9:1460. doi: 10.3389/fonc.2019.01460 (PMC6960125; doi:10.3389/fonc.2019.01460)
Supplement: Table S2 — Search strategies of electronic databases. [file Table_2.DOCX]

**Table S2** Search strategies of electronic databases

The combined text and medical subject heading (MeSH) terms used were: “small-cell lung cancer”.

**PubMed**

The database was searched on July 5, 2019，n=169

Search Strategy:

("once daily" OR "once a day" OR "one time daily" OR "one time a day") AND ("twice daily" OR "twice a day" OR "two times daily" OR "two times a day") AND (small cell lung carcinoma[MeSH Terms] OR small-cell lung carcinoma[Text Word] OR small cell lung cancer[MeSH Terms] OR small-cell lung cancer[Text Word] OR SCLC[Text Word] OR ((small cell) AND (lung)))

**Web of Science**

The database was searched on July 5, 2019，n=151

Search Strategy:

("small cell lung carcinoma" OR "small-cell lung cancer" OR "SCLC" OR ((small cell) AND (lung))) AND ("once daily" OR "once a day" OR "one time daily" OR "one time a day") AND ("twice daily" OR "twice a day" OR "two times daily" OR "two times a day")

**EMBASE**

The database was searched on July 5, 2019，n=246

Search Strategy:

('once daily' OR 'once a day' OR 'one time daily' OR 'one time a day') AND ('twice daily' OR 'twice a day' OR 'two times daily' OR 'two times a day') AND ('small cell lung carcinoma' OR 'small cell lung cancer' OR 'sclc' OR ((small cell) AND (lung)))

**Cochrane Library**

The database was searched on July 5, 2019，n=55

Search Strategy:

("small cell lung carcinoma" OR "small-cell lung cancer" OR "SCLC" OR ((small cell) AND (lung))):ti,ab,kw AND ("once daily" OR "once a day" OR "one time daily" OR "one time a day"):ti,ab,kw AND ("twice daily" OR "twice a day" OR "two times daily" OR "two times a day"):ti,ab,kw AND (radiotherapy):ti,ab,kw

**Scopus**

The database was searched on July 5, 2019，n=140

Search Strategy:

("small cell lung carcinoma" OR "small-cell lung cancer" OR "SCLC" OR ((small cell) AND (lung))) AND ("once daily" OR "once a day" OR "one time daily" OR "one time a day") AND ("twice daily" OR "twice a day" OR "two times daily" OR "two times a day")

**Ovid MEDLINE**

The database was searched on July 5, 2019，n=572

Search Strategy:

1.("small cell lung carcinoma" or "small-cell lung cancer" or "SCLC").mp. [mp=ab, hw, kw, ti, ot, tx, bt, ct, sh, sa, nm, fx, kf, ox, px, rx, an, ui, ds, on, sy]（136409）

2.radiotherapy.mp. [mp=ab, hw, kw, ti, ot, tx, bt, ct, sh, sa, nm, fx, kf, ox, px, rx, an, ui, ds, on, sy]（529722）

3.("once daily" or "once a day" or "one time daily" or "one time a day").mp. [mp=ab, hw, kw, ti, ot, tx, bt, ct, sh, sa, nm, fx, kf, ox, px, rx, an, ui, ds, on, sy]（229586）

4.("twice daily" or "twice a day" or "two times daily" or "two times a day").mp. [mp=ab, hw, kw, ti, ot, tx, bt, ct, sh, sa, nm, fx, kf, ox, px, rx, an, ui, ds, on, sy]（290644）

1 and 2 and 3 and 4(572)

**ScienceDirect**

The database was searched on July 5, 2019，n=160

Search Strategy:

Title, abstract, keywords: ("small cell lung carcinoma" OR "small-cell lung cancer" OR "SCLC" OR ((small cell) AND (lung))) AND ("once daily" OR "once a day" OR "one time daily" OR "one time a day") AND ("twice daily" OR "twice a day" OR "two times daily" OR "two times a day")

**Google Scholar**

The database was searched on July 5, 2019，n=6

Search Strategy:

("small cell lung carcinoma" OR "small-cell lung cancer" OR "SCLC" OR ((small cell) AND (lung))) AND ("once daily" OR "once a day" OR "one time daily" OR "one time a day") AND ("twice daily" OR "twice a day" OR "two times daily" OR "two times a day")
