# Supplementary material for: A Meta-Analysis of the Efficacy and Toxicity of Twice-Daily vs. Once-Daily Concurrent Chemoradiotherapy for Limited-Stage Small Cell Lung Cancer Based on Randomized Controlled Trials
Source: Front Oncol. 2020 Jan 8;9:1460. doi: 10.3389/fonc.2019.01460 (PMC6960125; doi:10.3389/fonc.2019.01460)
Supplement: Table S3 — GRADE quality assessment by therapeutic strategy and study design for the outcomes of survival and toxicity. [file Table_3.DOCX]

**Table S3** GRADE quality assessment by therapeutic strategy and study design for the outcomes of survival and toxicity

| **Primary outcomes** | **No. of Studies** | **No. of participants** | | **Differences^a^（95%CI）** | **Quality assessment** | | | | | **Quality** |
| --- | --- | --- | --- | --- | --- | --- | --- | --- | --- | --- |
|  |  | **BID** | **OD** |  | **Risk of bias^b^** | **Inconsistency** | **Indirectness** | **Imprecision** | **Publication bias^c^** |  |
| **Survival** |  |  |  |  |  |  |  |  |  |  |
| OS | 4 | 687 | 691 | 0.88 [0.78, 0.99] | Low | No inconsistency | No indirectness | No imprecision | Unlikely | High |
| OSR |  |  |  |  |  |  |  |  |  |  |
| 1-year | 4 | 549/687 | 519/691 | 1.07 [1.01, 1.13] | Low | No inconsistency | No indirectness | No imprecision | Unlikely | High |
| 2-year | 4 | 358/687 | 326/691 | 1.11 [1.00, 1.23] | Low | No inconsistency | No indirectness | No imprecision | Unlikely | High |
| 3-year | 4 | 256/687 | 236/691 | 1.09 [0.95, 1.26] | Low | No inconsistency | No indirectness | No imprecision | Unlikely | High |
| 4-year | 4 | 221/687 | 183/691 | 1.22 [1.03, 1.43] | Low | No inconsistency | No indirectness | No imprecision | Unlikely | High |
| 5-year | 4 | 199/687 | 169/691 | 1.19 [1.00, 1.41] | Low | No inconsistency | No indirectness | No imprecision | Unlikely | High |
| PFS | 3 | 476 | 458 | 0.92 [0.79, 1.07] | Low | No inconsistency | No indirectness | No imprecision | Unlikely | High |
| PFSR |  |  |  |  |  |  |  |  |  |  |
| 1-year | 2 | 138/203 | 144/215 | 1.03 [0.91, 1.17] | Low | No inconsistency | No indirectness | No imprecision | Unlikely | High |
| 2-year | 2 | 90/203 | 83/215 | 1.17 [0.94, 1.46] | Low | No inconsistency | No indirectness | No imprecision | Unlikely | High |
| 3-year | 2 | 65/203 | 64/215 | 1.09 [0.82, 1.45] | Low | No inconsistency | No indirectness | No imprecision | Unlikely | High |
| 4-year | 2 | 53/203 | 52/215 | 1.09 [0.78, 1.52] | Low | No inconsistency | No indirectness | No imprecision | Unlikely | High |
| 5-year | 2 | 49/203 | 52/215 | 1.01 [0.72, 1.41] | Low | No inconsistency | No indirectness | No imprecision | Unlikely | High |
| ORR | 2 | 235/269 | 238/269 | 0.99 [0.93, 1.05] | Low | No inconsistency | No indirectness | No imprecision | Unlikely | High |
| CR | 3 | 224/399 | 175/401 | 1.31 [1.01, 1.70] | Low | Serious (-1) | No indirectness | No imprecision | Unlikely | Moderate |
| PR | 2 | 101/269 | 136/269 | 0.76 [0.63, 0.92] | Low | No inconsistency | No indirectness | No imprecision | Unlikely | High |
| **Toxicity** |  |  |  |  |  |  |  |  |  |  |
| All grades AEs |  |  |  |  |  |  |  |  |  |  |
| Total | 1 | 204/206 | 202/203 | 1.00 [0.98, 1.01] | Low | No inconsistency | No indirectness | No imprecision | Likely (-1) | Moderate |
| Myelotoxicity | 1 | 199/206 | 201/203 | 0.98 [0.95, 1.00] | Low | No inconsistency | No indirectness | No imprecision | Likely (-1) | Moderate |
| Leukopenia | 1 | 197/206 | 190/203 | 1.02 [0.98, 1.07] | Low | No inconsistency | No indirectness | No imprecision | Likely (-1) | Moderate |
| Fatigue | 1 | 243/266 | 247/263 | 0.97 [0.93, 1.02] | Low | No inconsistency | No indirectness | No imprecision | Likely (-1) | Moderate |
| Neutropenia | 2 | 421/472 | 396/466 | 1.05[1.00, 1.10] | Low | No inconsistency | No indirectness | No imprecision | Unlikely | High |
| Anaemia | 2 | 401/472 | 390/466 | 1.02 [0.96, 1.07] | Low | No inconsistency | No indirectness | No imprecision | Unlikely | High |
| Oesophagitis | 2 | 365/460 | 315/499 | 1.19 [0.75, 1.90] | Low | Serious (-1) | No indirectness | No imprecision | Unlikely | Moderate |
| Nausea | 1 | 195/266 | 197/263 | 0.98 [0.88, 1.08] | Low | No inconsistency | No indirectness | No imprecision | Likely (-1) | Moderate |
| Thrombocytopenia | 1 | 134/206 | 125/203 | 1.06 [0.91, 1.22] | Low | No inconsistency | No indirectness | No imprecision | Likely (-1) | Moderate |
| Weight loss | 1 | 136/206 | 118/203 | 1.14 [0.98, 1.32] | Low | No inconsistency | No indirectness | No imprecision | Likely (-1) | Moderate |
| Anorexia | 1 | 153/266 | 150/263 | 1.01 [0.87, 1.17] | Low | No inconsistency | No indirectness | No imprecision | Likely (-1) | Moderate |
| Grade3-5 AEs |  |  |  |  |  |  |  |  |  |  |
| Total | 2 | 255/336 | 230/335 | 1.17 [0.80, 1.72] | Low | Serious (-1) | No indirectness | No imprecision | Unlikely | Moderate |
| Myelotoxicity | 1 | 179/206 | 173/203 | 1.02 [0.94, 1.10] | Low | No inconsistency | No indirectness | No imprecision | Likely (-1) | Moderate |
| Leukopenia | 3 | 340/409 | 334/419 | 1.04 [0.98, 1.11] | Low | No inconsistency | No indirectness | No imprecision | Likely (-1) | Moderate |
| Neutropenia | 3 | 422/545 | 395/550 | 1.06 [0.95, 1.17] | Low | Serious (-1) | No indirectness | No imprecision | Unlikely | Moderate |
| Thrombocytopenia | 3 | 129/409 | 155/419 | 0.86 [0.72, 1.03] | Low | No inconsistency | No indirectness | No imprecision | Unlikely | High |
| Oesophagitis | 5 | 157/675 | 121/691 | 1.39 [0.91, 2.11] | Low | Serious (-1) | No indirectness | No imprecision | Unlikely | Moderate |
| Anaemia | 4 | 109/675 | 100/682 | 1.10[0.86, 1.40] | Low | No inconsistency | No indirectness | No imprecision | Unlikely | High |
| Infection | 4 | 91/675 | 97/682 | 0.99[0.77, 1.26] | Low | No inconsistency | No indirectness | No imprecision | Unlikely | High |
| Nausea | 2 | 45/396 | 48/395 | 0.94[0.60, 1.37] | Low | No inconsistency | No indirectness | No imprecision | Unlikely | High |
| Fatigue | 1 | 31/266 | 31/263 | 0.99[0.62, 1.58] | Low | No inconsistency | No indirectness | No imprecision | Likely (-1) | Moderate |
| Vomiting | 3 | 52/602 | 50/598 | 1.04[0.72, 1.50] | Low | No inconsistency | No indirectness | No imprecision | Unlikely | High |
| TRM | 4 | 16/682 | 17/689 | 0.96[0.50, 1.87] | Low | No inconsistency | No indirectness | No imprecision | Unlikely | High |
| Pulmonary effects | 2 | 8/682 | 6/689 | 1.36 [0.49, 3.80] | Low | No inconsistency | No indirectness | No imprecision | Unlikely | High |
| Infection | 2 | 3/336 | 2/335 | 1.39 [0.28, 6.99] | Low | No inconsistency | No indirectness | No imprecision | Unlikely | High |
| Neutropenic sepsis | 1 | 1/273 | 3/270 | 0.33 [0.03, 3.15] | Low | No inconsistency | No indirectness | No imprecision | Likely (-1) | Moderate |
| Bronchial pneumonia | 1 | 0/273 | 2/270 | 0.20 [0.01, 4.10] | Low | No inconsistency | No indirectness | No imprecision | Likely (-1) | Moderate |
| Myelotoxicity | 1 | 1/206 | 0/203 | 2.96 [0.12, 72.15] | Low | No inconsistency | No indirectness | No imprecision | Likely (-1) | Moderate |
| Dementia | 1 | 0/273 | 1/270 | 0.33 [0.01, 8.06] | Low | No inconsistency | No indirectness | No imprecision | Likely (-1) | Moderate |
| Septic shock | 1 | 1/273 | 0/270 | 2.97 [0.12, 72.52] | Low | No inconsistency | No indirectness | No imprecision | Likely (-1) | Moderate |
| Peripheral vascular ischaemia | 1 | 1/273 | 0/270 | 2.97 [0.12, 72.52] | Low | No inconsistency | No indirectness | No imprecision | Likely (-1) | Moderate |

**Abbreviations:** BID: twice-daily; OD: once-daily; OS: overall survival; OSR: overall survival rate; PFS: progression-free survival; PFSR: progression-free survival rate; ORR: objective response rate; AEs: adverse effects; TRM: treatment-related mortality; CR: complete response; PR: partial response; CI: confidence interval.

^a^ Differences: hazard ratio (HR) for OS and PFS; risk ratio (RR) for OSR, PFSR, ORR, CR, PR, all grades AEs, grade3-5 AEs and TRM.

^b^ Risk of bias assessed using the Cochrane Collaboration’s tool for randomized controlled trials.

^c^ Publication bias was assessed by Egger and Begg tests.
