# Supplementary material for: A Meta-Analysis of the Efficacy and Toxicity of Twice-Daily vs. Once-Daily Concurrent Chemoradiotherapy for Limited-Stage Small Cell Lung Cancer Based on Randomized Controlled Trials
Source: Front Oncol. 2020 Jan 8;9:1460. doi: 10.3389/fonc.2019.01460 (PMC6960125; doi:10.3389/fonc.2019.01460)
Supplement: Table S4 — Ranking of the cause of treatment-related mortality. [file Table_4.DOCX]

**Table S4** Ranking of the cause of treatment-related mortality

| **Cause of**  **treatment-related death** | **Twice-daily arm (event/total)** | **Once-daily arm (event/total)** | **The incidence of adverse effects (%)^a^** | **RR (95% CI)** | ***P* value** | **Heterogeneity** | |
| --- | --- | --- | --- | --- | --- | --- | --- |
|  |  |  |  |  |  | ***I^2^* (%)** | ***P* value** |
| **Pulmonary effects** | 8/682 | 6/689 | 1.02 | 1.36 [0.49, 3.80] | 0.56 | 16 | 0.31 |
| **Infection** | 3/336 | 2/335 | 0.75 | 1.39 [0.28, 6.99] | 0.69 | 0 | 0.55 |
| **Neutropenic sepsis** | 1/273 | 3/270 | 0.74 | 0.33 [0.03, 3.15] | 0.34 | - | - |
| **Bronchial pneumonia** | 0/273 | 2/270 | 0.37 | 0.20 [0.01, 4.10] | 0.29 | - | - |
| **Myelotoxicity^b^** | 1/206 | 0/203 | 0.24 | 2.96 [0.12, 72.15] | 0.51 | - | - |
| **Dementia^c^** | 0/273 | 1/270 | 0.18 | 0.33 [0.01, 8.06] | 0.50 | - | - |
| **Septic shock** | 1/273 | 0/270 | 0.18 | 2.97 [0.12, 72.52] | 0.50 | - | - |
| **Peripheral vascular ischaemia** | 1/273 | 0/270 | 0.18 | 2.97 [0.12, 72.52] | 0.50 | - | - |

**Abbreviations**: RR: risk ratio; CI: confidence interval.

^a^: Event (twice-daily arm + once-daily arm)/Total (twice-daily arm + once-daily arm);

^b:^ **Myelotoxicity** was defined as any decrease in marrow-derived cells in the peripheral-blood counts;

^c:^ **Dementia** possibly related to prophylactic cranial irradiation.
